# 2819. Clinical Outcomes of Fosfomycin Treatment in *E. coli* vs. Non-*E. coli* Enterobacterales Urinary Tract Infection

**DOI:** 10.1093/ofid/ofad500.2430

**Published:** 2023-11-27

**Authors:** Kristi Traugott, Grace Lee, Lindsey Groff, Samantha Aguilar

**Affiliations:** University Health, San Antonio, Texas; The University of Texas at Austin College of Pharmacy, Pharmacotherapy Education and Research Center, School of Medicine, UT Health San Antonio, San Antonio, Texas; University Health, San Antonio, Texas; University Health, San Antonio, Texas

## Abstract

**Background:**

Oral fosfomycin tromethamine is considered a first-line agent for the treatment of uncomplicated acute cystitis in women according to the national Infectious Disease Society of America (IDSA) guideline. However, non-*E. coli* Enterobacterales urinary tract infection (UTI) pathogens exhibit higher minimum inhibitory concentrations (MICs) for fosfomycin and limited data exist to determine whether treatment outcomes are comparable to patients being treated with fosfomycin for *E.coli* UTIs. The objective of this study is to compare the clinical outcomes of patients being treated with fosfomycin who have *E. coli* UTIs compared to non-*E. coli* Enterobacterales UTIs.

**Methods:**

This was a retrospective noninferiority study of adult inpatients with a UTI who received a fosfomycin inpatient from July 15, 2020 to March 15, 2023. The primary outcome was incidence of treatment failure within 30 days of start of fosfomycin, defined by retreatment for UTI; UTI related- hospitalization, urgent care visits, and/or ambulatory visits; subsequent positive blood culture; and/or infection related mortality. One-sided test for noninferiority was used with a 20% margin. Multivaraible logistic regression analyses were conducted adjusting for age/sex, UTI severity, and prior UTIs.

**Results:**

A total of 77 patients were included; 45 with *E. coli* UTIs and 32 with non-*E. coli* Enterobacterales UTIs. The majority of the patients were female (80.5%), with a median age of 67 years (IQR, 61-69), Hispanic/Latino (50.6%), had chronic kidney disease (33%), and diabetes mellitus (35%), and were admitted to a non-ICU floor (79%). The overall incidence of treatment failure was 19.5% (90% CI 12.9%-27.7%); 15.6% in those with *E. coli* vs. 25% in those with non-*E. coli* Enterobacterales UTIs (p=0.385; absolute difference 9.4%; 90% CI -7.3% to 26.2%), not meeting the criteria for non-inferiority. In multivariable analysis, *E. coli* vs. non-*E. coli* Enterobacterales UTIs did not associate with treatment failure (adjusted odds ratio, 0.66; 95% CI 0.193-2.25; p=0.505).

**Figure:**
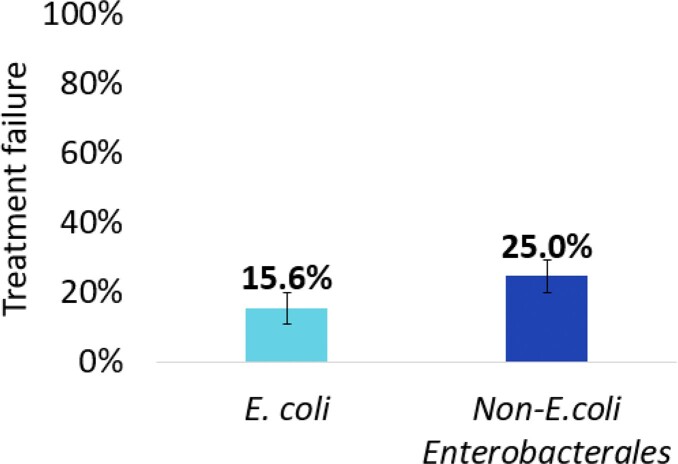

**Conclusion:**

Fosfomycin treatment for non-E. coli Enterobacterales UTIs did not meet non-inferiority criteria when compared to patients with E. coli UTIs. Further studies with larger sample sizes are required.

**Disclosures:**

**All Authors**: No reported disclosures

